# Comparison of the Pharmacokinetic Properties of Triamcinolone and Dexamethasone for Local Therapy of the Inner Ear

**DOI:** 10.3389/fncel.2019.00347

**Published:** 2019-07-30

**Authors:** Alec Nicholas Salt, Jared James Hartsock, Jennifer Hou, Fabrice Piu

**Affiliations:** ^1^Department of Otolaryngology, Washington University School of Medicine, St. Louis, MO, United States; ^2^Otonomy Inc., San Diego, CA, United States

**Keywords:** intratympanic therapy, Meniere’s disease, idiopathic sudden sensorineural hearing loss, dexamethasone, triamcinolone, triamcinolone acetonide

## Abstract

Some forms of triamcinolone may provide alternate options for local therapy of the inner ear in addition to the steroids currently in use. We compared the perilymph pharmacokinetics of triamcinolone-acetonide, triamcinolone, and dexamethasone, each delivered as crystalline suspensions to guinea pigs. Triamcinolone-acetonide is a widely used form of the drug with molecular properties that allow it to readily permeate biological barriers. When applied intratympanically triamcinolone-acetonide entered perilymph rapidly but was also found to be eliminated rapidly from perilymph. The rapid rate of elimination severely limits the apical distribution of the drug when applied locally, making it unsuitable for use in the ear. In contrast, triamcinolone, rather than triamcinolone-acetonide, is a more polar form of the molecule, with higher aqueous solubility but calculated to pass less-readily through biological boundaries. Perilymph concentrations generated with intratympanic applications of triamcinolone were comparable to those with triamcinolone-acetonide but elimination measurements showed that triamcinolone was retained in perilymph longer than triamcinolone-acetonide or dexamethasone. The slower elimination is projected to result in improved distribution of triamcinolone toward the cochlear apex, potentially allowing higher drug levels to reach the speech frequency regions of the human ear. These measurements show that triamcinolone could constitute an attractive additional treatment option for local therapy of auditory disorders.

## Introduction

Treatment of the inner ear with a locally applied steroid has become a widely accepted therapy for Meniere’s disease, idiopathic sudden sensorineural hearing loss and immune-related hearing loss. The choice of which steroid and what concentration to use has largely been established empirically based except for a limited few pioneering studies where drug kinetics (dexamethasone and methylprednisolone) were compared in animals (Parnes et al., 1999) and in humans (Bird et al., 2007, 2011). Currently, steroids that have been used for local inner ear therapy include dexamethasone-phosphate (Liebau et al., 2017); dexamethasone (Lambert et al., 2016); methylprednisolone-succinate (Rauch et al., 2011; Liebau et al., 2017); and triamcinolone-acetonide (Jumaily et al., 2017; Dahm et al., 2019).

We recently reported that dexamethasone-phosphate, the most common form of dexamethasone used for local treatment of the ear, has properties that make it quite unsuitable for local therapy of the ear (Salt et al., 2018). The polar phosphate group that makes the drug more soluble also makes this form of the drug substantially less permeable though the RW membrane than native dexamethasone, limiting the amount entering. Within the ear it is then metabolized to the active form, dexamethasone, which is highly permeable through the blood-labyrinth barrier and is rapidly lost from perilymph. The high rate of dexamethasone elimination limits how far the drug spreads apically along the ear (Plontke et al., 2008; Salt et al., 2018). As a result, concentrations reaching the speech frequency regions of the human are calculated to be low (Liebau et al., 2017). Large gradients along the ear will persist even when higher doses of dexamethasone-phosphate are given (Alexander et al., 2015).

A second problem exists when the steroid is applied to the middle ear as a solution. For dexamethasone-phosphate, loss from the middle ear occurs rapidly. Measurement of the solution remaining in the RW niche of guinea pigs demonstrated a decline in concentration with a half-time of approximately 28 min (Salt et al., 2018). This rate occurred in anesthetized recumbent animals which did not lose volume through the Eustachian tube. The rate of loss is likely to be even higher in the conscious animal in normal posture in which fluid in the middle ear is cleared by the ciliated ventral epithelium to the Eustachian tube (Thompson and Tucker, 2013). The residence time of drug in the middle ear has a major influence on the perilymph concentration of drug achieved (Salt and Plontke, 2005). A longer drug residence time in the middle ear can be achieved by delivering the drug as a suspension. Measurable perilymph concentrations of dexamethasone were achieved for over 3 months after intratympanic application of dexamethasone suspension (Wang et al., 2009; Piu et al., 2011). Both triamcinolone-acetonide and triamcinolone exhibit low aqueous solubility making them suitable for delivery to the middle ear as a suspension. We therefore evaluated the pharmacokinetics of different formulations these two compounds in perilymph and compared them with comparable measurements with dexamethasone which were previously reported (Salt et al., 2012, 2018).

**FIGURE 1:**
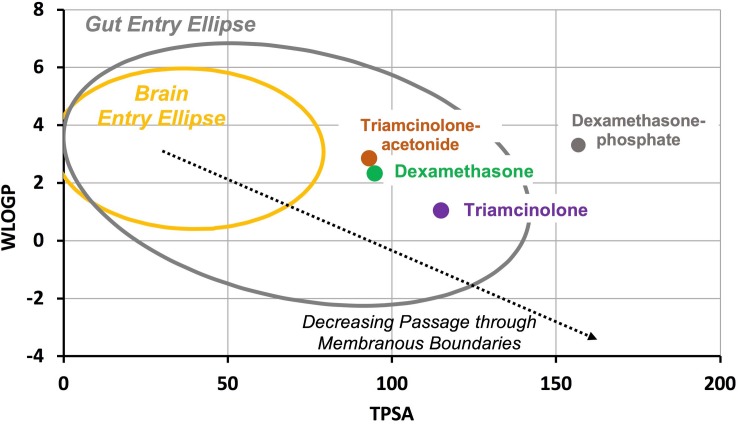
Molecular properties compared for common forms of dexamethasone and triamcinolone. WLOGP is a calculated index of lipid solubility for the drug and TPSA represents the surface area of the molecule occupied by polar groups. Large, polar, hydrophilic molecules (lower right of plot) typically have high aqueous solubility but do not readily pass through membranous biological boundaries. In contrast, small, non-polar lipophilic molecules (upper left of plot) pass more readily through biological boundaries but have lower aqueous solubility. The yellow ellipse indicates the range of properties for molecules that pass through the blood brain barrier and the gray ellipse indicates the range of properties for molecules that pass into the body through the gut (Daina and Zoete, 2016). Dexamethasone-phosphate is larger and more polar than dexamethasone, giving it higher aqueous solubility but limiting passage through biological boundaries. Triamcinolone-acetonide is calculated to pass boundaries more readily than dexamethasone while triamcinolone is calculated to pass less readily.

The ability of molecules to pass through biological barriers such as the blood-brain barrier and the gut has been shown to depend on their lipid solubility and polar properties (Egan et al., 2000; Daina and Zoete, 2016). A plot of lipid solubility (WLOGP) against the polar surface area (TPSA) shows that molecules with properties within a specific range can pass certain biological boundaries, as shown in Figure 1. The gut is permeable to molecules within a specific range (gray ellipse) while the blood-brain barrier is more restrictive, only permeable to molecules within a more limited range (yellow ellipse) (Daina and Zoete, 2016). The degree to which inner ear barriers, specifically those between the middle ear and perilymph and between perilymph and blood, compare to these better-studied boundaries remains to be determined. Nevertheless, molecules with properties located toward the lower right side of the plot (large, hydrophilic, and polar) are expected to pass through membranous biological boundaries less easily than molecules located toward upper left side of the plot (small, lipophilic, and non-polar). The plot shows the molecular properties calculated by SwissADME^1^ (Daina et al., 2017) for triamcinolone-acetonide and triamcinolone, compared to those of dexamethasone-phosphate and dexamethasone. Triamcinolone-acetonide is more lipophilic than dexamethasone while triamcinolone is more polar and less lipophilic.

## Materials and Methods

### Animals

The study used 32 pigmented, NIH-strain guinea pigs weighing 400–600 g. Experiments were performed under protocol 20160053, approved by the Institutional Animal Care and Use Committee of Washington University. Animal use followed policies in accordance with the United States Department of Agriculture and National Institutes of Health guidelines for the handling and use of laboratory animals.

In non-recovery perilymph sampling experiments, animals were first anesthetized with 100 mg/kg sodium thiobutabarbital (Inactin, Sigma, St. Louis, MO, United States). A tracheal cannula was inserted and the animal was maintained on 0.8 to 1.2% isoflurane in oxygen using a mechanical ventilator. End-tidal CO_2_ level was maintained close to 5% by adjustment of the tidal volume of the ventilator. Heart rate and oxygen saturation were monitored throughout the experiment. Core body temperature was maintained at 38°C with a thermistor-controlled heating blanket.

The ear was exposed by opening the lateral portion of the auditory bulla. This gave access to the LSCC and to the RW niche. Drug solutions were either applied to the RW niche or injected into perilymph of the lateral SCC. In all experiments, perilymph was sampled from the lateral SCC. In some experiments as detailed below, triamcinolone-acetonide or triamcinolone suspension was applied intratympanically as a recovery procedure, followed by perilymph sampling 1 or 3 days afterward.

### Drug Delivery: Application to the Round Window Niche

In non-recovery experiments, the lateral bulla was opened and suspensions of triamcinolone-acetonide or triamcinolone were applied to the RW niche with a hand-held micropipetter. A 20 μL volume was applied, sufficient to fill the entire niche and stapes area of the guinea pig, with excess overflowing toward the anterior bulla.

Drug formulations were:

(1) Triamcinolone-acetonide 40 mg/mL injectable crystalline suspension (Kenalog-40: Bristol-Myers Squibb, Princeton, NJ, United States). Kenalog-40 also contains 0.99% benzyl alcohol as a preservative (in higher concentrations shown to increase permeability of the RW membrane and stapes; Li et al., 2018) and 0.04% polysorbate-80 (Tween-80), an emulsifier. This formulation was applied to two animals. The aqueous solubility of triamcinolone-acetonide is reported to be 42 μg/mL (Drugbank)^2^. We measured aqueous solubility by taking a 1:1 dilution of the supernatant over the suspension after it had settled for at least 24 h. The concentration in the supernatant, corrected for dilution, was 26.5 μg/mL; *SD* 1.7; *n* = 4.

(2) Triamcinolone-acetonide (Spectrum Chemicals, New Brunswick, NJ, United States) 60 mg/mL crystalline suspension in PBS with 17% Poloxamer 407 (Spectrum Chemicals). This formulation was applied to five animals.

(3) Triamcinolone (Sigma, St. Louis, MO, United States) 40 mg/mL suspension in PBS with 17% Poloxamer 407. This formulation was applied to 12 animals. Aqueous solubility of triamcinolone is reported to be 80 μg/mL (PubChem)^3^. The concentration of the supernatant over the suspension was measured to be 81.05 μg/mL; *SD* 29.4; *n* = 4).

### Drug Delivery: Intratympanic Injection

The procedure for intratympanic injection of drug suspension in poloxamer gel is described elsewhere in detail (Salt et al., 2011). The guinea pig was anesthetized with isoflurane (0.8–1.2% in oxygen) and buprenorphine (0.05 mg/kg) given for analgesia. The head of the animal was positioned against a foam pad on a head-holder with its nose vertically upward, held in position by a Velcro strap. A speculum was placed in the right ear, allowing the tympanic membrane to be visualized. A hypodermic needle held in a manipulator was used to make two perforations in the tympanic membrane, one anterior to the umbo as a vent and a second posterior to the umbo for injection. Drug injection through the latter perforation was performed from a blunt-tipped 26G needle, bent 90 degrees and attached to a syringe mounted on a syringe pump (Ultrapump; World Precision Instruments, Sarasota, FL).

Perilymph was sampled from the LSCC either 1 or 3 days after the application of the drug formulation. Triamcinolone-acetonide suspension was applied in three animals followed by perilymph sampling 1 day later. Triamcinolone suspension was applied in 6 animals followed by perlilymph sampling 1 day (*n* = 4) or 3 days (*n* = 2) later.

### Drug Delivery: Injection Into the Lateral Semi-Circular Canal

The rates of elimination of triamcinolone or triamcinolone-acetonide from perilymph were measured by first loading the perilymph with drug solution as detailed in Salt et al. (2012). At varying times after application (zero, 1 or 2 h) perilymph was sampled from the LSCC for analysis to establish how much drug remained in perilymph. The injected solution consisted of the supernatant over the suspension as described above, diluted 1:1 in a bicarbonate-buffered artificial perilymph. Triamcinolone-acetonide solution was applied in four animals and triamcinolone solution was applied in 12 animals.

### Sequential Sampling From the Lateral Semi-Circular Canal

Sequential sampling of perilymph makes it possible to quantify drug concentration gradients along the perilymphatic spaces (Mynatt et al., 2006; Salt et al., 2012). The LSCC was prepared for perilymph sampling by first removing the mucosa and cleaning the bone and with saline and cotton swabs. The bone overlying the canal was thinned with a dental burr and a thin layer of cyanoacrylate glue (Permabond 101; Permabond, Pottstown, PA, United States) was applied. A covering of two-part silicone adhesive (Kwik-Cast, World Precision Instruments, Sarasota, FL, United States), was applied to this surface, built up at the edges to form a hydrophobic cup. This allowed perilymph to be collected free of contamination of drug solution in the rest of the bulla.

Sampling commenced by making a 30–40 μm fenestration in the silicone-coated canal wall with a 30° House stapes pick (N1705 80, Bausch and Lomb Inc.). The perilymph emerging from the perforation accumulated on the hydrophobic surface of the silicone where it was collected in hand-held, blunt tipped capillary tubes (VWR 53432-706), marked to a nominal volume of 1 μL. Twenty individual samples were collected from each animal, each taking 1–2 min. The exact volume of each sample was determined by measuring the sample length under a calibrated dissecting microscope. The 20 samples were paired consecutively and each pair added to 50 μL of 1:1 methanol:water diluent, resulting in 10 diluted samples, each containing 2 μL perilymph and 50 μL diluent. Samples were stored frozen at −80°C until analysis.

### Analysis of Samples

Samples were analyzed using high-pressure liquid chromatography combined with mass spectrometry detection (Wang et al., 2011). HPLC operators were blinded to the experimental conditions and to the origin of the samples. The LLOQ for triamcinolone was 0.2–1 ng/mL and the LLOQ for triamcinolone-acetonide was 0.28 ng/mL. In experiments where triamcinolone was applied to the ear only triamcinolone was measured. In experiments where triamcinolone-acetonide was applied, both triamcinolone-acetonide and triamcinolone were measured.

### Quantitative Simulation of Sequential Sample Data

A computer program simulating the inner ear fluids (which can be downloaded from)^4^ can be set to replicate all aspects of the experiments performed in this study in great detail. For intratympanic applications, calculations include known entry sites from the middle ear into perilymph of the guinea pig, including the RW, stapes and through the thin bone at the apex. Drug distribution through the fluid and tissue spaces of the entire inner ear is calculated based on defined diffusion coefficients and elimination rates. Simulation of drug injections into the lateral SCC and the sequential sampling procedure takes into account the induced volume flows. In the perilymph sampling procedure, the specific volumes and collection times for each of the samples are replicated. Rates of elimination were adjusted to best fit the calculated sample concentrations from the model to the measured sample data, allowing the kinetic properties of the drugs to be compared quantitatively.

## Results

### Triamcinolone-Acetonide Kinetics

**FIGURE 2:**
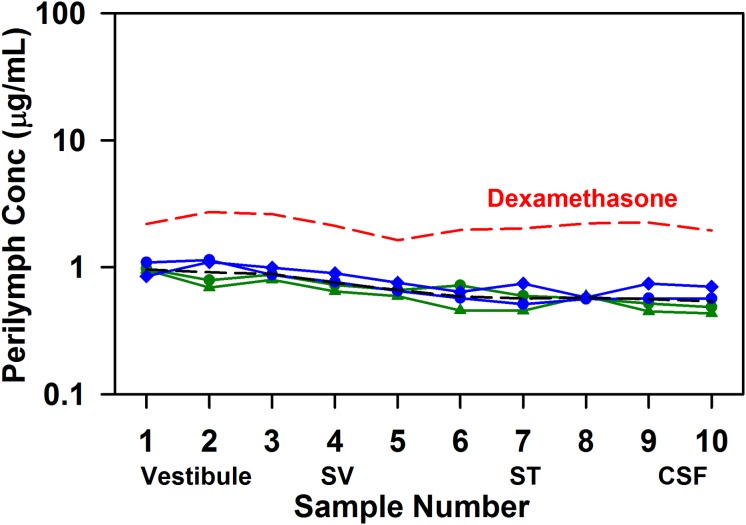
Perilymph triamcinolone-acetonide concentrations achieved 1 h after application of triamcinolone-acetonide suspension to the RW niche of guinea pigs. Perilymph was collected as 10 × 2 uL sequential samples from the LSCC, each of which was analyzed independently. Early samples originate from the vestibule, followed by SV and ST in sequence. In two experiments the drug suspension was applied as a liquid (green curves) and in two experiments in poloxamer gel (blue curves), with similar results. The group mean (four experiments) is shown as a black dotted line. For comparison the group mean results of similar experiments applying dexamethasone suspension (Salt et al., 2018) are shown as the red dotted line.

Triamcinolone-acetonide suspension was applied to the RW niche for 60 min before perilymph was sampled. Figure 2 shows the perilymph levels measured when the triamcinolone-acetonide suspension was applied as a liquid (green) or gel (blue) formulation. Perilymph concentrations were found to be lower than found in previous studies with a dexamethasone suspension (red dashed line; from Salt et al., 2018). It is important to note that these are results using dexamethasone suspension, which differ considerably from the commonly used solution of dexamethasone-phosphate. The average across all 40 samples for triamcinolone-acetonide was 0.71 ug/mL compared to 2.16 ug/mL for dexamethasone. Although perilymph triamcinolone-acetonide concentrations were lower than those for dexamethasone by a factor of 3.1×, this is largely explained by differences in the middle ear concentration of the two drugs. Triamcinolone-acetonide has 3.6× lower aqueous solubility (26.5 ug/mL) compared to dexamethasone (94.2 ug/mL), so there will be a lower concentration driving entry into perilymph. Although both triamcinolone and triamcinolone-acetonide were measured in the samples, the vast majority of the drug was present in perilymph as triamcinolone-acetonide. In 37 of the 40 samples triamcinolone concentration was unmeasurable, but in three of the samples a low concentration of triamcinolone was present, averaging just 1.4% of the total drug concentration.

**FIGURE 3:**
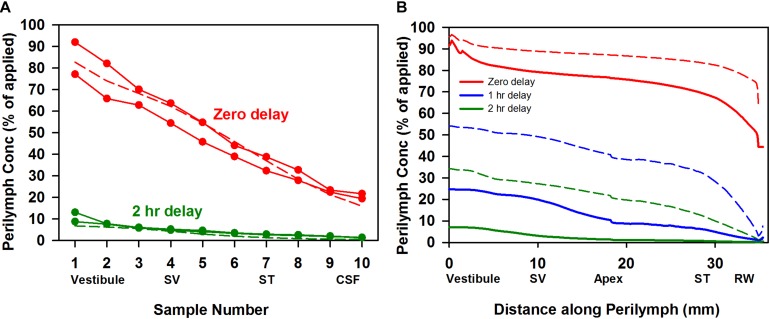
**(A)** Elimination measurement of triamcinolone-acetonide. Solid lines with symbols are individual experiments in which triamcinolone-acetonide concentrations of perilymph samples obtained by sequential sampling were measured either immediately (zero delay; red curves, *n* = 2) or 2 h (green curves, *n* = 2) after perilymph loading. Dotted lines show calculated sample concentrations derived by simulation with elimination half time 12 min for ST and 34 min for SV, parameters which best fit the data set. **(B)** Solid lines show the calculated distribution of triamcinolone-acetonide along the perilymphatic space at zero (red), 1 h (blue), and 2 h (green) delay after loading calculated using the same kinetic parameters. For comparison curves based on a best fit to dexamethasone sampling data are shown dotted (ST elimination: 46 min; SV elimination 91 min; Salt et al., 2018). Triamcinolone-acetonide is lost more rapidly from perilymph than dexamethasone, faster than any other substance measured to date.

In separate experiments the rate of triamcinolone-acetonide elimination from perilymph was quantified by loading perilymph by injection from a pipette sealed into the lateral SCC. Figure 3A shows the measured perilymph concentrations from four experiments, two sampled immediately after loading and two measured at 2 h after loading. Concentrations shown are the sum of triamcinolone-acetonide and triamcinolone in each sample. These studies show that triamcinolone-acetonide elimination occurred extremely rapidly, falling to 5–10% of the applied concentration within 2 h. The declining curve for perilymph seen with zero delay time was also consistent with rapid elimination. It shows the injection rate was insufficient to load the ear uniformly with drug, due to the high ongoing elimination rate during injection. Simulations fitted to the perilymph measurements provide the most quantitative way to compare kinetics across drugs. Elimination rates that best-fitted the measured data (dotted lines) were half times of 34 min in SV and 12 min in ST. These are the most rapid rates of drug elimination from perilymph we have ever measured. Figure 3B (solid lines) shows the calculated perilymph distribution of triamcinolone-acetonide at the three sampling times. Also shown on the plot for comparison (dotted lines) is the calculated distribution for dexamethasone at the same times, using a ST elimination half-time of 46 min and a SV elimination half-time of 91 min (Salt et al., 2018). Elimination of triamcinolone-acetonide occurs far faster than that of dexamethasone. The metabolism of triamcinolone-acetonide to triamcinolone was also apparent in the data from canal injection experiments, with triamcinolone making up 10.5% of the total in zero delay experiments and 63.3% of the total after 2 h. The accumulation of triamcinolone proportion with time provided the first indication that triamcinolone was better retained in perilymph than triamcinolone-acetonide.

### Triamcinolone Kinetics

**FIGURE 4:**
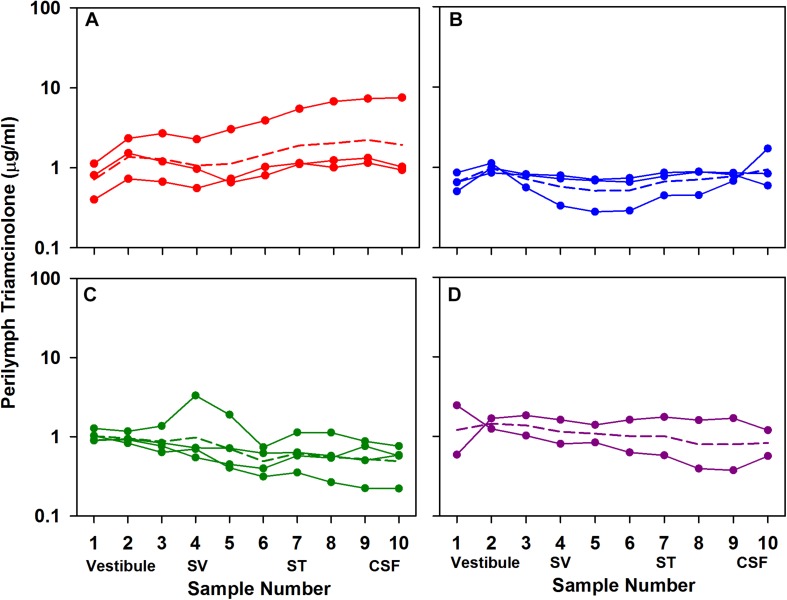
Perilymph triamcinolone concentrations measured 1 h **(A)**, 3 h **(B)**, 1 day **(C)**, and 3 days **(D)** after application of triamcinolone suspension to the RW niche of guinea pigs. Perilymph was collected as 10×2 μL sequential samples each of which was analyzed independently. Each curve shows the measured data from a single animal. Animal numbers in each group were 3, 3, 4, and 2, respectively. For each group the mean is shown as a dotted line.

A suspension of triamcinolone was applied to the RW niche either in acute experiments with sampling 1 or 3 h after application to the RW niche, or as recovery experiments with sampling 1 or 3 days after intratympanic injection. The measured perilymph concentrations are summarized in Figure 4. A relatively uniform perilymph concentration over the time period was observed. Even after 3 days, triamcinolone crystals were visible in the RW niche and the perilymph concentration was maintained. The average concentrations for each of the groups (averaging all samples) were 1.52 μg/mL (*n* = 3; 1 h); 0.71 μg/mL (*n* = 3; 3 h); 0.72 (*n* = 4; 1 day); and 1.07 (*n* = 2, 3 days). Significance testing with two way ANOVA indicated that perilymph levels at 1 h were significantly higher than at 3 h and 1 day (Bonferroni, *P* < 0.001). It is also notable that the perilymph concentrations of triamcinolone were quite similar to those observed above with triamcinolone-acetonide, and even more so when normalized for middle ear concentration differences for the two forms of triamcinolone.

**FIGURE 5:**
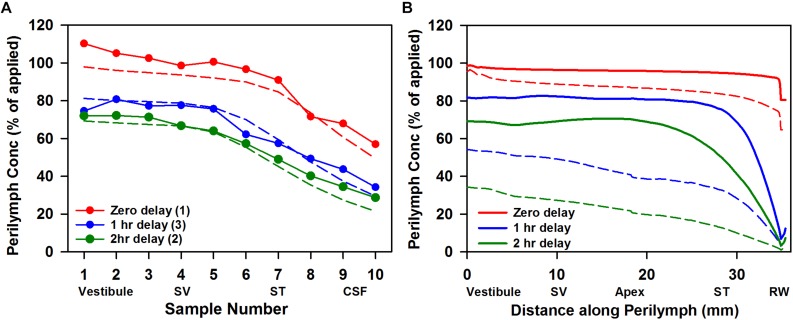
**(A)** Elimination measurement of triamcinolone. Solid lines with symbols are the group means of the number of experiments indicated in which triamcinolone concentrations of perilymph samples obtained by sequential sampling were measured either immediately (zero delay; red curve, *n* = 1), 1 h (blue curve, *n* = 3), or 2 h (green curve, *n* = 2) after perilymph loading. Dotted lines show calculated sample concentrations derived by simulation with elimination half time 700 min for ST and 278 min for SV; parameters which best fit this data set. **(B)** Solid lines show the distribution of triamcinolone along the perilymphatic space at zero, 1 and 2 h delay after loading calculated using the same parameters. For comparison, curves calculated based on a best fit to dexamethasone sampling data are shown dotted (ST elimination: 46 min; SV elimination 91 min; Salt et al., 2018).

The rate of elimination of triamcinolone from perilymph was measured by loading perilymph by injection from a pipette sealed into the LSCC. The results of six experiments in which perilymph was sampled after zero delay (*n* = 1), 1 hr delay (*n* = 3); or 2 h delay (*n* = 2) are summarized in Figure 5. The data show that triamcinolone is retained well in perilymph, with initial vestibular samples at 70% of the applied drug concentration after 2 h. Dotted lines show the best fit of the kinetic model to the data set, with elimination half times of 278 min in SV and 700 min in ST. In Figure 5B the calculated distribution of triamcinolone along the perilymphatic spaces for the three time periods (solid lines) is compared with the distribution of dexamethasone (dotted lines). It is apparent that triamcinolone is retained in perilymph much better than triamcinolone-acetonide and dexamethasone, consistent with the more polar properties of this form of the molecule.

**FIGURE 6:**
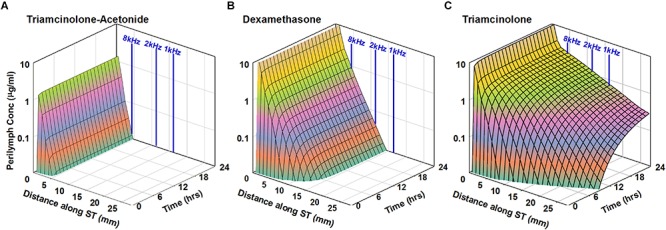
Simulation of intratympanic applications of triamcinolone-acetonide suspension **(A)**, dexamethasone suspension **(B)**, and triamcinolone suspension **(C)** to the human ear. Entry and elimination kinetics are based on perilymph measurements in guinea pigs. ST elimination half-times were 12, 44, and 700 min for triamcinolone-acetonide, dexamethasone, and triamcinolone, respectively. Triamcinolone-acetonide and dexamethasone only distribute apically to a limited degree before reaching a steady state, unlike triamcinolone which reaches the cochlear apex.

Based on the kinetic parameters for entry into perilymph from the middle ear and elimination from perilymph to the vasculature derived in this study, we are able to project how the two forms of triamcinolone might compare with the distribution of dexamethasone in the human cochlea. In each case, the drug can be applied as a suspension so the concentration in the middle ear is assumed constant over the 24 h period calculated, with the dissolved (free) concentration based on the aqueous solubility of the compound. The calculated perilymph concentrations as a function of distance along the cochlea and of time are shown in Figure 6.

As triamcinolone-acetonide is lost very rapidly from perilymph it will not spread far apically before being lost. Dexamethasone distributes further along the cochlea but does not reach apical regions in appreciable concentration. In contrast triamcinolone is calculated to distribute throughout the length of the human cochlea as a result of the lower rate of elimination from perilymph.

## Discussion

The elimination of locally applied drugs from perilymph has a major influence on effective local drug therapy of the inner ear. If elimination occurs more rapidly than the drug diffuses along the scala, this may limit the distribution of the drug along the cochlea. The influence of elimination also depends on the cochlear dimensions of the species under study. For a given drug, concentration gradients along the ear as a result of elimination will be substantially smaller in short cochleas, such as those of mice, and greater in longer cochleas, such as those of humans.

Measurement of elimination from perilymph was performed here by loading the fluid and tissue spaces of the inner ear with drug and then following the subsequent decline of drug with time by taking fluid samples at varying delay times after loading. This method overcomes difficulties with other application methods where drug gradients in the ear may exist. If drug gradients exist, decline of drug concentration due to distribution into regions of lower concentration cannot be differentiated from the decline due to elimination. Triamcinolone-acetonide was shown to be rapidly eliminated from perilymph while triamcinolone had completely different characteristics and was retained well. Elimination of dexamethasone occurred at an intermediate rate. The measured elimination characteristics are consistent with the polar properties of the three molecules influencing passage across biological lipid membrane boundaries. In the case of elimination measurements, the molecular properties likely influence their ability to pass through cell membranes of the capillary endothelial cells (Salt and Hirose, 2018).

An interesting observation from this study is that while elimination differed markedly between triamcinolone-acetonide (fast) and triamcinolone (slow), the perilymph levels measured with RW niche applications were similar for both drugs. Simulations (not shown) indicate that perilymph concentration of drug depends on the rate of entry from the middle ear, balanced against the losses from perilymph due to elimination to blood and from interactions with cerebrospinal fluid. A similar perilymph level can result from fast entry with fast elimination (as in the case of triamcinolone-acetonide) or from slower entry with a slower rate of elimination (as in the case of triamcinolone). This raises an intriguing idea as it suggests that measured perilymph concentrations resulting from intratympanic applications cannot be easily interpreted in terms of the underlying perilymph pharmacokinetics. The similar perilymph levels observed here with triamcinolone and triamcinolone-acetonide gave no indication that the underlying kinetics in perilymph were completely different. In order to understand the perilymph kinetics additional measurements, such as measurement of elimination as performed here is required.

Our pharmacokinetic measurements suggest that triamcinolone should be considered as a potential therapy for auditory disorders where steroid therapy is indicated. Specifically, triamcinolone has pharmacokinetic properties that are predicted to allow it to reach apical cochlear regions of the human ear in higher concentration, potentially allowing all frequency regions to be treated. The choice of steroid for use in the clinic is, however, complex and based on many factors other than pharmacokinetic studies in animals. For instance, relative potencies at the target receptors are critical. Triamcinolone has been reported to have 660× lower potency than dexamethasone, while triamcinolone-acetonide may have 2–6× higher potency than dexamethasone at the glucocorticoid receptor (Mayer et al., 1974; Pratt et al., 1975; Yeakley et al., 1980; Grossmann et al., 2004; Nehmé et al., 2009). Potency estimates vary with many factors, however, including species, tissue and application duration (Giannopoulos and Keichline, 1981; Nehmé et al., 2009). Relative steroid potency remains unmeasured for tissues of the human inner ear. The relative affinity of mineralocorticoid receptors to the different steroids is also unknown. Another major factor concerns the difference between the concentrations of steroid in the tissues compared to those in perilymph and the factors contributing to tissue absorption. Although dexamethasone time courses have been compared in tissues and plasma with systemic dosing (Cherlet et al., 2005; Li et al., 2017) and in vitreous humor and retina tissues for the eye with local delivery (Chang-Lin et al., 2011), neither of these systems exhibit concentration gradients comparable to those seen in the ear. The cochlear tissue concentrations of steroid resulting from different perilymph distributions therefore remain unknown. It is possible that for some therapies of the ear, such as the treatment of Meniere’s disease, the spatial distribution of drug may not be a limiting factor and treatment of vestibular and basal cochlear tissue may be sufficient. In animals, triamcinolone-acetonide therapy.

So in some cases, there may be limited benefit to a more apical distribution of drug along the cochlea. It therefore becomes important to evaluate how patients with auditory disorders respond to triamcinolone therapy. Unfortunately, human pilot studies with triamcinolone have been hampered by the lack of an available formulation approved for intravenous use, as exists for triamcinolone-acetonide. As triamcinolone-acetonide has higher potency for systemic delivery it has replaced the use of triamcinolone, so comparable formulations of triamcinolone are no longer available. Nevertheless, for the treatment of auditory disorders, a drug which can provide apical regions with substantially higher concentration would seem desirable and potentially allow all frequency regions of the human ear to be treated.

The three drugs compared here were all delivered to the middle ear as crystalline suspensions, which dissolve slowly over a period of weeks. Unlike solutions, where drug is lost rapidly from the middle ear, suspensions provide a depot effect, increasing the duration of the drug time course in perilymph. Such a “depot,” influence has previously been reported for both triamcinolone-acetonide (Ye et al., 2007; Honeder et al., 2014) and for dexamethasone (Wang et al., 2009, 2011).

Our studies show that the requirements for an effective steroid for inner ear therapy are very different from those for intravenous or oral therapies. With local therapy the ease of passage through membranous boundaries, such as the RW membrane and blood-perilymph barrier, influence the spatial distribution of drug. The physical properties of the molecule that determine membrane permeability (such as lipophilicity and polarity) therefore play an important role in the cochlear distribution of drug. With systemic delivery, the molecular form is of lesser importance if the drug is metabolized to an active form in organs such as the liver. We have previously shown that the molecular properties of dexamethasone-phosphate make it quite unsuitable for local therapy of the ear (Salt et al., 2018). The present study shows that triamcinolone-acetonide, a more potent form of triamcinolone in widespread clinical use, also does not have appropriate kinetic properties for local treatment of the ear. In contrast, the more polar triamcinolone appears better suited. We conclude that the properties that make a drug suitable for local use in the ear are completely different from those that make the drug suitable for intravenous or oral use.

A key factor to improve drug distribution along the length of the ear is to reduce the rate of elimination. The triamcinolone molecule is almost identical to dexamethasone, differing by one non-polar CH_3_ group in dexamethasone which is replaced by a polar OH group in triamcinolone. As can be seen in our measurements, this modest increase in polar properties markedly increases retention in perilymph, thus predicting the drug to be distributed more evenly along the ear. One possible confounding factor could have been that the reduced passage through membranous boundaries would limit entry from the middle ear. The perilymphatic measurements with local applications showed that any reduction in entry from the middle ear is largely offset by reduced rate of elimination, so that the measured perilymph concentration of triamcinolone was similar to that of triamcinolone-acetonide (Figures 2, 4).

## Conclusion

In conclusion, the studies described herein suggest that administration to the middle ear of a triamcinolone suspension yields an attractive pharmacokinetic profile in the perilymph. These measurements show that triamcinolone could provide an additional treatment option for local therapy of auditory disorders.

## Data Availability

All datasets generated for this study are included in the manuscript and/or the supplementary files.

## Ethics Statement

Experiments were performed under protocol 20160053, approved by the Institutional Animal Care and Use Committee of Washington University. Animal use followed policies in accordance with the United States Department of Agriculture and National Institutes of Health guidelines for the handling and use of laboratory animals.

## Author Contributions

AS and FP conceived and designed the study. JJH and JH collected, organized, and analyzed the data. AS wrote the first draft of the manuscript. All authors contributed to manuscript revision, read, and approved the submitted version.

## Conflict of Interest Statement

AS is a paid consultant to Otonomy, Inc. FP and JH are employees of Otonomy, Inc. This project was not funded by Otonomy. Other projects in AS’s laboratory are funded by Cochlear Corp. and Frequency Therapeutics, Inc. The remaining author declares that the research was conducted in the absence of any commercial or financial relationships that could be construed as a potential conflict of interest.

## Footnotes

1 http://www.swissadme.ch

2 https://www.drugbank.ca/

3 http://pubchem.ncbi.nlm.nih.gov

4 http://oto.wustl.edu/saltlab

## Abbreviations

LSCC: lateral semi-circular canal
RW: round window
ST: scala tympani
SV: scala vestibuli.

## References

[B1] Alexander T. H. Harris J. P. Nguyen Q. T. Vorasubin N. (2015). Dose effect of intratympanic dexamethasone for idiopathic sudden sensorineural hearing Loss: 24 mg/mL Is Superior to 10 mg/mL. *Otol. Neurotol.* 36 1321–1327. 10.1097/MAO.0000000000000834 26196209

[B2] Bird P. A. Begg E. J. Zhang M. Keast A. T. Murray D. P. Balkany T. J. (2007). Intratympanic versus intravenous delivery of methylprednisolone to cochlear perilymph. *Otol. Neurotol.* 28 1124–1130. 10.1097/mao.0b013e31815aee21 18043438

[B3] Bird P. A. Murray D. P. Zhang M. Begg E. J. (2011). Intratympanic versus intravenous delivery of dexamethasone and dexamethasone sodium phosphate to cochlear perilymph. *Otol. Neurotol.* 32 933–936. 10.1097/MAO.0b013e3182255933 21725263

[B4] Chang-Lin J. E. Attar M. Acheampong A. A. Robinson M. R. Whitcup S. M. Kuppermann B. D. (2011). Pharmacokinetics and pharmacodynamics of a sustained-release dexamethasone intravitreal implant. *Invest Ophthalmol. Vis. Sci.* 52 80–86. 10.1167/iovs.10-5285 20702826

[B5] Cherlet M. De Baere S. Croubels S. De Backer P. (2005). Quantitative determination of dexamethasone in bovine plasma and tissues by liquid chromatography–atmospheric pressure chemical ionization–tandem mass spectrometry. *Analytica Chimica Acta* 529 361–369. 10.1016/j.aca.2004.07.014

[B6] Dahm V. Nieratschker M. Riss D. Kaider A. Auinger A. Honeder C. (2019). Intratympanic triamcinolone acetonide as treatment option for idiopathic sudden sensorineural hearing loss. *Otol. Neurotol.* 40 720–727. 10.1097/MAO.0000000000002283 31192900

[B7] Daina A. Michielin O. Zoete V. (2017). SwissADME: a free web tool to evaluate pharmacokinetics, drug-likeness and medicinal chemistry friendliness of small molecules. *Sci. Rep.* 7:42717. 10.1038/srep42717 28256516 PMC5335600

[B8] Daina A. Zoete V. (2016). A BOILED-Egg To predict gastrointestinal absorption and brain penetration of small molecules. *ChemMedChem* 11 1117–1121. 10.1002/cmdc.201600182 27218427 PMC5089604

[B9] Egan W. J. Merz K. M. Jr. Baldwin J. J. (2000). Prediction of drug absorption using multivariate statistics. *J. Med. Chem.* 43 3867–3877. 10.1021/jm000292e 11052792

[B10] Giannopoulos G. Keichline D. (1981). Species-related differences in steroid-binding specificity of glucocorticoid receptors in lung. *Endocrinology.* 108 1414–1419. 10.1210/endo-108-4-1414 7472274

[B11] Grossmann C. Scholz T. Rochel M. Bumke-Vogt C. Oelkers W. Pfeiffer A. F. (2004). Transactivation via the human glucocorticoid and mineralocorticoid receptor by therapeutically used steroids in CV-1 cells: a comparison of their glucocorticoid and mineralocorticoid properties. *Eur. J. Endocrinol.* 151 397–406. 10.1530/eje.0.1510397 15362971

[B12] Honeder C. Engleder E. Schöpper H. Gabor F. Reznicek G. Wagenblast J. (2014). Sustained release of triamcinolone acetonide from an intratympanically applied hydrogel designed for the delivery of high glucocorticoid doses. *Audiol. Neurootol.* 19 193–202. 10.1159/000358165 24714604 PMC4717230

[B13] Jumaily M. Faraji F. Mikulec A. A. (2017). Intratympanic triamcinolone and dexamethasone in the treatment of ménière’s syndrome. *Otol. Neurotol. Mar.* 38 386–391. 10.1097/mao.0000000000001311 28192380

[B14] Lambert P. R. Carey J. Mikulec A. A. LeBel C. (2016). Otonomy ménière’s study group. intratympanic sustained-exposure dexamethasone thermosensitive gel for symptoms of ménière’s disease: randomized phase 2b safety and efficacy trial. *Otol. Neurotol.* 37 1669–1676. 10.1097/mao.0000000000001227 27749754 PMC5414596

[B15] Li W. Hartsock J. J. Dai C. Salt A. N. (2018). Permeation enhancers for intratympanically-applied drugs studied using fluorescent dexamethasone as a marker. *Otol. Neurotol.* 39 639–647. 10.1097/MAO.0000000000001786 29649043 PMC5940507

[B16] Li X. DuBois D. C. Song D. Almon R. R. Jusko W. J. Chen X. (2017). Modeling combined immunosuppressive and anti-inflammatory effects of dexamethasone and naproxen in rats predicts the steroid-sparing potential of naproxen. *Drug Metab Dispos.* 45 834–845. 10.1124/dmd.117.075614 28416614 PMC5469402

[B17] Liebau A. Pogorzelski O. Salt A. N. Plontke S. K. (2017). Hearing changes after intratympanically applied steroids for primary therapy of sudden hearing loss: a meta-analysis using mathematical simulations of drug delivery protocols. *Otol. Neurotol.* 38 19–30. 10.1097/mao.0000000000001254 27779563 PMC5154844

[B18] Mayer M. Kaiser N. Milholland R. J. Rosen F. (1974). The binding of dexamethasone and triamcinolone acetonide to glucocorticoid receptors in rat skeletal muscle. *J. Biol. Chem.* 249 5236–5240. 4369267

[B19] Mynatt R. Hale S. A. Gill R. M. Plontke S. K. R. Salt A. N. (2006). Demonstration of a longitudinal concentration gradient along scala tympani by sequential sampling of perilymph from the cochlear apex. *J. Assoc. Res. Otolaryngol.* 7 182–193. 10.1016/j.jneumeth.2005.10.008 16718612 PMC1945159

[B20] Nehmé A. Lobenhofer E. K. Stamer W. D. Edelman J. L. (2009). Glucocorticoids with different chemical structures but similar glucocorticoid receptor potency regulate subsets of common and unique genes in human trabecular meshwork cells. *BMC Med. Genomics* 2:58. 10.1186/1755-8794-2-58 19744340 PMC2749862

[B21] Parnes L. S. Sun A. H. Freeman D. J. (1999). Corticosteroid pharmacokinetics in the inner ear fluids: an animal study followed by clinical application. *Laryngoscope.* 109 1–17. 10.1097/00005537-199907001-00001 10399889

[B22] Piu F. Wang X. Fernandez R. Dellamary L. Harrop A. Ye Q. (2011). OTO-104: a sustained-release dexamethasone hydrogel for the treatment of otic disorders. *Otol. Neurotol.* 32 171–179. 10.1097/MAO.0b013e3182009d29 21099726

[B23] Plontke S. K. Biegner T. Kammerer B. Delabar U. Salt A. N. (2008). Dexamethasone concentration gradients along scala tympani after application to the round window membrane. *Otol. Neurotol.* 29 401–406. 10.1097/mao.0b013e318161aaae 18277312 PMC2587453

[B24] Pratt W. B. Kaine J. L. Pratt D. V. (1975). The kinetics of glucocorticoid binding to the soluble specific binding protein of mouse fibroblasts. *J. Biol. Chem.* 250 4584–4591. 166997

[B25] Rauch S. D. Halpin C. F. Antonelli P. J. Babu S. Carey J. P. Gantz B. J. (2011). Oral vs intratympanic corticosteroid therapy for idiopathic sudden sensorineural hearing loss: a randomized trial. *JAMA* 305 2071–2079. 10.1001/jama.2011.679 21610239

[B26] Salt A. N. Hartsock J. J. Gill R. M. Piu F. Plontke S. K. (2012). Perilymph pharmacokinetics of markers and dexamethasone applied and sampled at the lateral semi-circular canal. *J. Assoc. Res. Otolaryngol.* 13 771–783. 10.1007/s10162-012-0347-y 22968908 PMC3505589

[B27] Salt A. N. Hartsock J. J. Piu F. Hou J. (2018). Dexamethasone and dexamethasone phosphate entry into perilymph compared for middle ear applications in guinea Pigs. *Audiol. Neurootol.* 23 245–257. 10.1159/000493846 30497073 PMC6492027

[B28] Salt A. N. Hartsock J. J. Plontke S. K. LeBel C. Piu F. (2011). Distribution of dexamethasone and preservation of inner ear function following intratympanic delivery of a gel-based formulation. *Audiol. Neurotol.* 16 323–335. 10.1159/000322504 21178339 PMC3023000

[B29] Salt A. N. Hirose K. (2018). Communication pathways to and from the inner ear and their contributions to drug delivery. *Hear Res.* 362 25–37. 10.1016/j.heares.2017.12.010 29277248 PMC5911243

[B30] Salt A. N. Plontke S. K. (2005). Local inner-ear drug delivery and pharmacokinetics. *Drug Discov. Today.* 10 1299–1306. 10.1016/s1359-6446(05)03574-9 16214674 PMC2681268

[B31] Thompson H. Tucker A. S. (2013). Dual origin of the epithelium of the mammalian middle ear. *Science.* 22 1453–1456. 10.1126/science.1232862 23520114

[B32] Wang X. Dellamary L. Fernandez R. Harrop A. Keithley E. M. Harris J. P. (2009). Dose-dependent sustained release of dexamethasone in inner ear cochlear fluids using a novel local delivery approach. *Audiol. Neurootol.* 14 393–401. 10.1159/000241896 19923809

[B33] Wang X. Dellamary L. Fernandez R. Ye Q. LeBel C. Piu F. (2011). Principles of inner ear sustained release following intratympanic administration. *Laryngoscope.* 121 385–391. 10.1002/lary.21370 21271594

[B34] Ye Q. Tillein J. Hartmann R. Gstoettner W. Kiefer J. (2007). Application of a corticosteroid (*Triamcinolon*) protects inner ear function after surgical intervention. *Ear. Hear.* 28 361–369. 10.1097/01.aud.0000261655.30652.62 17485985

[B35] Yeakley J. M. Balasubramanian K. Harrison R. W. (1980). Comparison of glucocorticoid-receptor binding kinetics with predictions from a biomolecular model. *J. Biol. Chem.* 255 4182–4188. 7372674

